# The benefits and pitfalls of machine learning for biomarker discovery

**DOI:** 10.1007/s00441-023-03816-z

**Published:** 2023-07-27

**Authors:** Sandra Ng, Sara Masarone, David Watson, Michael R. Barnes

**Affiliations:** 1 Centre for Translational Bioinformatics, William Harvey Research Institute, Queen Mary University of London, London, UK; 2 Alan Turing Institute, London, UK; 3 Department of Informatics, King’s College London, London, UK

**Keywords:** Machine learning, Artificial intelligence, Biomarker, Transcriptomics, Multi-omics

## Abstract

Prospects for the discovery of robust and reproducible biomarkers have improved considerably with the development of sensitive omics platforms that can enable measurement of biological molecules at an unprecedented scale. With technical barriers to success lowering, the challenge is now moving into the analytical domain. Genome-wide discovery presents a problem of scale and multiple testing as standard statistical methods struggle to distinguish signal from noise in increasingly complex biological systems. Machine learning and AI methods are good at finding answers in large datasets, but they have a tendency to overfit solutions. It may be possible to find a local answer or mechanism in a specific patient sample or small group of samples, but this may not generalise to wider patient populations due to the high likelihood of false discovery. The rise of explainable AI offers to improve the opportunity for true discovery by providing explanations for predictions that can be explored mechanistically before proceeding to costly and time-consuming validation studies. This review aims to introduce some of the basic concepts of machine learning and AI for biomarker discovery with a focus on post hoc explanation of predictions. To illustrate this, we consider how explainable AI has already been used successfully, and we explore a case study that applies AI to biomarker discovery in rheumatoid arthritis, demonstrating the accessibility of tools for AI and machine learning. We use this to illustrate and discuss some of the potential challenges and solutions that may enable AI to critically interrogate disease and response mechanisms.

## Introduction

A biomarker is a measurable characteristic that reflects the physiological or pathological state of an organism. These characteristics can be genes, proteins, metabolic pathways etc., and they can be used to diagnose diseases, monitor treatment outcomes or predict disease progression. Biomarker discovery has been revolutionised by omics technologies that allow high-throughput profiling of biological molecules in cells and tissues in all range of states and conditions. These platforms are capable of measuring millions of features, including, just to give a few examples, genotype, epigenetic state and the levels of RNA, proteins and metabolites. This capability for discovery has shifted investigation towards large collaborative projects and resources where sample size can also be maximised to improve power and reduce cost at scale (Box 1). While omics platforms are readily accessible to researchers, their relatively high cost per sample and, more importantly, the logistics of large-scale experimentation — particularly collection of sufficient patient samples — can still present substantial limitations on experimental design and analysis.

Box 1 Key examples of genetic and genomic biomarker discovery resources.

**Table:** 

The Cancer Genome Atlas (TCGA) is a comprehensive and coordinated effort to accelerate our understanding of the molecular basis of cancer through the application of genome analysis technologies, including large-scale genome sequencing. It includes data from over 11,000 patients across 33 different cancer types (Cancer Genome Atlas Research Network et al. 2013)
The Encyclopedia of DNA Elements (ENCODE) project is a large-scale research effort aimed at identifying and characterising functional elements in the human genome (ENCODE Project Consortium 2012). The ENCODE project has generated a large amount of data, including over 1400 datasets from over 4000 experiments in a wide range of samples, including over 150 cell lines and primary cells, and over 150 tissues, including the brain, heart, liver, kidney, lung and blood. Omics platforms include: • ChIP-seq (chromatin immunoprecipitation followed by sequencing) for mapping protein-DNA interactions • RNA-seq (RNA sequencing) for transcriptome analysis • DNase-seq (DNAse I hypersensitivity sequencing) for identifying open chromatin regions • FAIRE-seq (formaldehyde-assisted isolation of regulatory elements) for identifying open chromatin regions
The Genome Aggregation Database (gnomAD) is a large-scale database of human genetic variation that includes data on over 150 million DNA variants from over 140,000 individuals from various ethnic groups, including African, African American, Ashkenazi Jewish, East Asian, Latino and Non-Finnish European populations. The data includes information on single-nucleotide variants (SNVs), small insertions and deletions (indels) and structural variants (SVs) (Karczewski et al. 2020)
The Human Microbiome Project (HMP) is a research effort to understand the microbial communities that live in and on the human body and how they affect health and disease. It includes data from over 300 individuals across five different body sites (Human Microbiome Project Consortium 2012)
The Human Protein Atlas (HPA) is a research project aimed at mapping all the human proteins in cells, tissues and organs using various omics technologies (Uhlén et al. 2015)

## Correlation or causation?

Biomarker discovery can help to form hypotheses, but, considered in isolation, biomarkers offer only limited mechanistic insight. This is exemplified by the use of C-reactive protein (CRP) as a biomarker of cardiovascular disease (CVD). CRP is a commonly used inflammatory biomarker, the high levels of which have been consistently linked to an increased risk of CVD. However, the exact nature of this relationship has long been disputed (Lagrand et al. 1999). It is possible that CRP levels are elevated as a result of CVD rather than being the cause of it. This creates confusion regarding the direction of the causal relationship between CRP and CVD. CRP levels can also be influenced by other factors such as obesity, physical activity and diet, which may confound signals between CRP and CVD still further. To clarify this association, temporal studies were performed following groups of individuals over time and observing changes in CRP levels and incidence of CVD. Conclusively, a later study found that elevated CRP levels preceded the onset of CVD events, suggesting that CRP levels may be a predictor of CVD risk rather than a consequence of the disease (Danesh et al. 2000).

## Defining endotypes

One approach to improved mechanistic understanding in biomarker discovery focuses on the concept of endotypes. Although patients may clinically manifest in a similar manner, an endotype defines a subgroup of patients who share a common underlying biology or pathway mechanism, which drives the manifestation of the disease or phenotype. Conceptually, biomarker discovery could also be seen as a way to pinpoint and extend endotypes. In the context of omics, by characterising the unique molecular signatures of different endotypes, it is possible to gain new insights into disease biology, to stratify patients and develop more targeted and effective treatments, ultimately bringing us ever closer to the goal of personalised medicine. The concept of the disease endotype was first defined in asthma (Lötvall et al. 2011), a chronic respiratory disease characterised by airway inflammation and bronchial hyperresponsiveness. Using transcriptomics, a range of immune/inflammatory endotypes has been defined in association with disease activity and therapeutic response (Shaw et al. 2015). Endotypes have been associated with a more severe form of asthma and a poorer response to conventional asthma treatments, with direct implications for the development of targeted and personalised treatment strategies (Shaw et al. 2015).

## Why is machine learning needed?

Biomarker measurements at the scale described above have obvious potential for innovative new discovery but are very challenging for long established statistical methods, such as *t*-tests and ANOVA. Many of these methods assume specific data distributions, such as normality, which is often not the case for data generated by genomic platforms. Deep sampling can produce complex data that defy any simple parametric description. For example, outliers and biomarkers beyond detection limits can skew a distribution, while natural phenomena like gene duplication, recombination and selection can lead to kurtosis, with highly peaked distributions of allele frequencies. As the computational complexity of analysis grows, it also becomes infeasible to run many conventional methods, as large omics datasets with potentially millions of features often contain nonlinear relationships. In addition to computational challenges, the form and structure of high dimensional datasets can be hard to visualise, making it difficult for humans to recognise or interpret patterns.

These challenges have all led to the development of a range of new machine learning (ML) methods that are able to deal with scale, diverse data distributions and non-linearity. ML algorithms are powerful tools for finding patterns in large datasets to predict outcomes or classify groups based on input data like transcriptomic profiles, with potential to improve our understanding of biology and enable personalised medical treatment based on a patient’s unique biomolecular profile.

ML is generally separated into supervised and unsupervised approaches.^1^ In the context of biomarker discovery, supervised ML involves training a model on a labelled dataset, where the input data (such as gene expression or proteomic measurements) and the output data (e.g. a disease diagnosis or prognosis) are known. The goal is to learn a mapping from inputs to outputs, so that the model can make predictions on new, unseen data. Unsupervised ML, on the other hand, involves training a model on an unlabelled dataset to uncover patterns or relationships without any prior knowledge or assumptions about the output. Common applications of unsupervised learning in biomarker discovery include clustering and dimensionality reduction techniques.

## A practical example of machine learning applied to transcriptomic data from RA patients

To illustrate many of the concepts discussed in this review, we have included a Python notebook containing a fully documented machine learning pipeline that includes many of the key steps described here. To achieve this, we used a public dataset exploring transcriptome expression in the blood of rheumatoid arthritis (RA) patients (Tasaki et al. 2018) (see https://github.com/C4TB/CTR_XAI). This notebook requires little to no coding expertise, and we encourage users of all backgrounds to explore. We have found the workbook framework to be quite flexible and easily modified to accommodate the user’s own data.

## Unsupervised learning

**Fig. 1:**
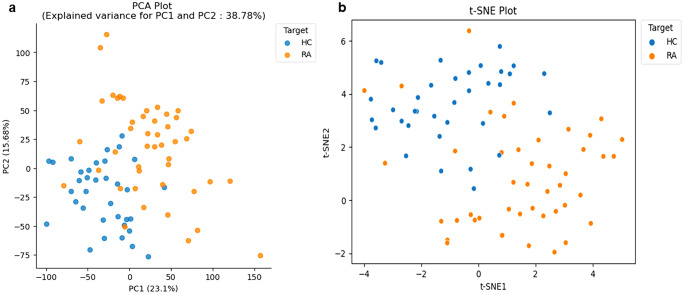
Unsupervised learning in RA using dimensionality reduction methods, **a** PCA and **b** t-SNE clustering labelled post hoc by disease activity

Unsupervised learning is often the first part of a bioinformatics analysis pipeline. Exploratory data analysis often begins with dimensionality reduction techniques such as principal component analysis (PCA), t-stochastic neighbour embedding (t-SNE), UMAP (Becht et al. 2019) and non-negative matrix factorisation (NMF) ((Lee and Seung 1999; Johnson et al. 2007; Van der Maaten and Hinton 2008). These techniques can help find structure in multidimensional data by reducing the complexity and projecting it onto a lower-dimensional space where group dynamics can become more salient and potential outliers can be more easily identified. These methods can also play an important role in data quality control (QC). In Fig. 1a and b, examples of PCA and t-SNE visualisations of the RA-patients are shown. The plots show quite clear separation and clustering of patients by disease status; notably, some patients cluster with controls and vice versa. These individual samples should probably be double checked to exclude laboratory errors, such as mislabelled samples.

Although both plots separate patient and control groups, they do so in different ways. PCA (Fig. 1a) aims to represent high-dimensional transcriptome data in a lower-dimensional space while preserving the total variation using a set of orthogonal linear projections. This provides a global view of the data, identifying the most important directions of change across the entire dataset, projected onto a new coordinate system defined by this variation. By contrast, t-SNE (Fig. 1b) aims to reduce dimensionality while preserving the local structure using nonlinear feature separation. This can be useful for identifying clusters, since the projection maintains pairwise similarities between samples. By transposing the input matrix, we can also use these methods to cluster features instead of samples, which can help to identify sets of co-expressed genes that are biologically relevant. These clusters can then be further analysed to gain insights into the functional relationships among the genes and their underlying biological processes.

Both methods have contrasting pros and cons. PCA is fast and computationally efficient, making it suitable for large datasets. The global view it provides identifies key sources of variation across the entire dataset, particularly where a strong linear relationship exists. However, PCA may not preserve the local structure of the data, making it difficult to identify subtle patterns or clusters of co-expressed genes that are biologically relevant. It also assumes that the data are normally distributed, which is often not the case in transcriptomic data. This makes PCA less robust to outliers and otherwise noisy or incomplete data.t-SNE is particularly useful for identifying local structure, e.g. clusters of co-expressed genes that are biologically relevant. The method is widely applied to single-cell transcriptome data. For example, in a single-cell RNAseq (scRNAseq) study of human skin, t-SNE was used to identify clusters of cells with similar gene expression patterns, leading to the discovery of new epidermal cell types and sub-populations (Joost et al. 2016). In the case of scRNAseq, t-SNE may not preserve the global structure of the data as well as PCA, making the latter more suited for preliminary clustering of single-cell transcriptomes, using genes that are informative across entire set of cells. In a scRNAseq study of the mouse brain, PCA was most suitable for finding major cell types, but t-SNE proved better at finding meaningful subdivisions among cells of the same kind (e.g. interneurons in a dataset containing both neurons, vascular cells and glia), leading to a loss of information about the major sources of variation (Zeisel et al. 2018). Some critics allege that t-SNE distorts the data in counterproductive ways (Chari and Pachter 2022), although it remains a popular visualisation tool for practitioners.

Like many other forms of machine learning, t-SNE performance can be dependent on the choice of hyperparameters, i.e. user-selected values chosen prior to analysis. Hyperparameters are often tuned using trial and error or systematic search methods to improve the performance of a machine learning model. Hyperparameters affect the behaviour of an algorithm and can significantly impact the model’s ability to learn and generalise to other data sets (Wattenberg et al. 2016).

Clustering algorithms are another large family of unsupervised learning methods. These may give very different outputs based on the underlying assumptions of the methods or the parameters used for analysis. This can create a problem where multiple clustering solutions are proposed. These can be evaluated by post hoc labelling with clinical features after the model has been trained. The results can be compared to known clinical features to see how well the model has captured meaningful patterns in the data. Ultimately, however, the aim of machine learning is to determine true structure in data and post hoc labelling could lead to confirmation bias (Prosperi et al. 2019).

A more rigorous approach to determine the most likely cluster structure is consensus clustering, a popular technique in which a clustering algorithm is trained on multiple subsets of the data to form more stable clusters (Monti 2003). The idea is that if two samples are co-clustered in all subsets in which both appear, then they are very likely to belong to the same group. This approach can potentially increase the robustness of a clustering solution and reduce the effect of random fluctuations in the data. There are different methods for combining and evaluating results across subsamples to form a consensus clustering solution, such as majority voting, co-association matrices, or inspecting the cumulative distribution function of co-clustering indices (John et al. 2020).

Overall, unsupervised learning can be a valuable tool for biomarker discovery because it allows exploration of data and identification of patterns or relationships that may not be obvious with other methods. It can also help to identify potential therapeutic targets or predictors of disease outcomes, even in the absence of clear underlying mechanisms or endotypes.

## Supervised machine learning

**Fig. 2:**
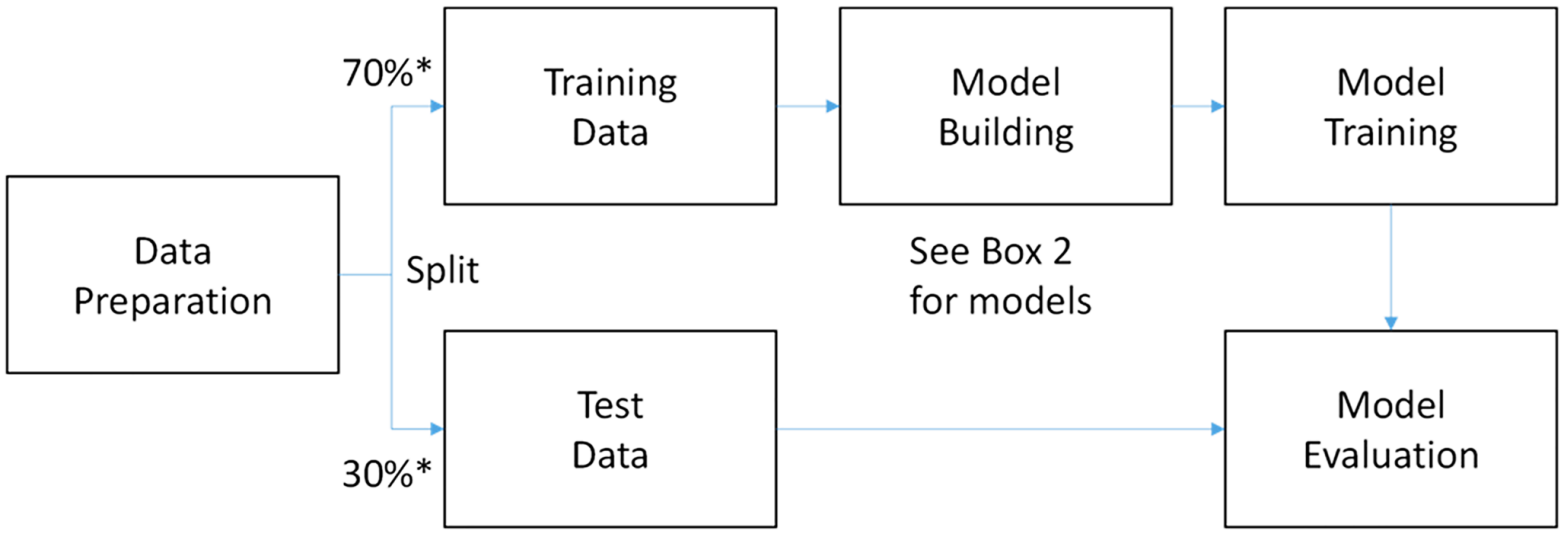
Supervised machine learning process overview. Starting with the raw input data, the process includes data preprocessing, feature extraction, model selection, model training, model validation and model deployment. The ultimate goal of this process is to develop a machine learning model that can accurately predict outcomes based on the input data. *Training/test split may be context dependent

Supervised ML algorithms are trained on labelled data to make predictions about new, unseen samples. Although these methods may differ quite widely, they typically involve a range of common steps (Fig. 2). Data pre-processing is required to remove noise, normalise the data and reduce its dimensionality (often using unsupervised methods). This is especially relevant for high-dimensional and noisy omics data. Labelled data is used to train the ML model, and this might involve selecting a subset of samples with known labels, such as the healthy versus diseased samples in our RA exemplar. There are many different types of supervised methods that can be used in omics analysis (see Box 2 for a summary with examples). The optimal model is selected based on performance metrics, such as accuracy or area under the receiver operating characteristic curve. The trained model is evaluated on a separate set of labelled data to estimate its predictive performance, which is important to ensure that the model is not overfitting to the training set. Once the model is trained and evaluated, it can be used to make predictions or classifications on new, unseen samples.

Supervised learning has many applications in omics analysis, including predicting patient outcomes, identifying biomarkers for disease and classifying disease subtypes. These methods are very well suited to these tasks, but ultimately the quality of the prediction is only as good as the data used to train it. The vast majority of omics biomarker studies are substantially limited by the quality and quantity of available data. Experiments should be guided by realistic power calculations to determine the number of samples required to detect an effect (Forshed 2017). When relevant effects are modest, thousands of patient samples may be needed. However, recruiting high numbers of patients is expensive and time-consuming. Moreover, while high-throughput sequencing costs have fallen dramatically since the technology was originally introduced, omics data generation is not cheap. Thus, compromises are often made in practice.

As shown in Fig. 2, data are split into training and test sets. A common convention is to use 70% of samples for training and 30% for testing. However, this split may not be optimal for all datasets and algorithms. The most suitable split percentage for training and test data in machine learning depends on the size and complexity of the dataset, the number of features and the specific machine learning algorithm being used. In general, larger datasets can benefit from a smaller percentage of training data, such as a 60/40 or 50/50 split, as they have more data to learn from.

Another alternative, which is preferable when data is scarce, is to use *k*-fold cross-validation. This involves randomly splitting the samples into *k* “folds” of approximately equal size. We then train *k* separate models, each excluding a single fold. Out of sample performance is then averaged across the folds. This way, each sample gets to be an element of a test set. Common values for *k* include 5 and 10. While cross-validation is more data efficient than the holdout method, it can be time-consuming to train a large number of complex models (Hastie et al. 2001).

In the context of transcriptomic and other omics data, several common supervised learning methods can be used to classify samples or predict clinical properties like disease activity or drug response (Box 2). These models and many others can be accessed with extensive exemplars and documentation in Scikit-learn, an open-source machine learning library for Python, which provides a wide range of tools for building and evaluating machine learning models (https://scikit-learn.org/).

Box 2 Key types of supervised machine learning used in genomic biomarker discovery studies. While linear and logistic regression are sometimes regarded as classical methods that fall outside the scope of ML, we emphasise that in high-dimensional settings, these models generally require regularisation penalties that take us beyond the realm of classical statistics. See Hastie et al. (2015).

**Table:** 

Method	References
**Linear regression:** this method can be used to model the relationship between transcriptomic features and a continuous outcome, such as disease activity score or surrogate marker of disease activity like blood lipid levels	Inouye et al. (2010)
**Logistic regression:** this method can be used to model the relationship between transcriptomic features and a binary outcome, such as the presence or absence of a disease. For example, Chanrion et al. used logistic regression to predict recurrence of tamoxifen-treated primary breast cancer based on gene expression	Chanrion et al. (2008); Chadeau-Hyam et al. (2013)
**Support vector machines (SVM)**: SVM is a kernel-based machine learning algorithm that can be used for classification or regression tasks with transcriptomic features	Huang et al. (2018)
**Random forest**: random forest is an ensemble machine learning method that creates many decision trees and combines their predictions to make a final prediction. It can be used for both regression and classification problems	Boulesteix et al. (2012)
**Gradient boosting:** this is an ensemble method that sequentially trains decision trees to improve upon the errors of the previous tree	Ma et al. (2020)
**Feedforward neural networks:** this is a type of neural network that consists of an input layer, one or more hidden layers and an output layer. The input layer takes the transcriptomic features as input, and the output layer produces a prediction, e.g. of disease activity	Yu et al. (2019)
**Convolutional neural networks (CNNs):** CNNs are a type of neural network that are particularly well suited for analysing image and other grid-structured data, such as genomic data	Yu et al. (2019)
**Recurrent neural networks (RNNs):** RNNs are a type of neural network that are designed to handle sequential data, such as time series. They have been applied to transcriptomic data for tasks such as gene expression prediction and disease diagnosis	Yu et al. (2019)

## Evaluating model performance

**Fig. 3:**
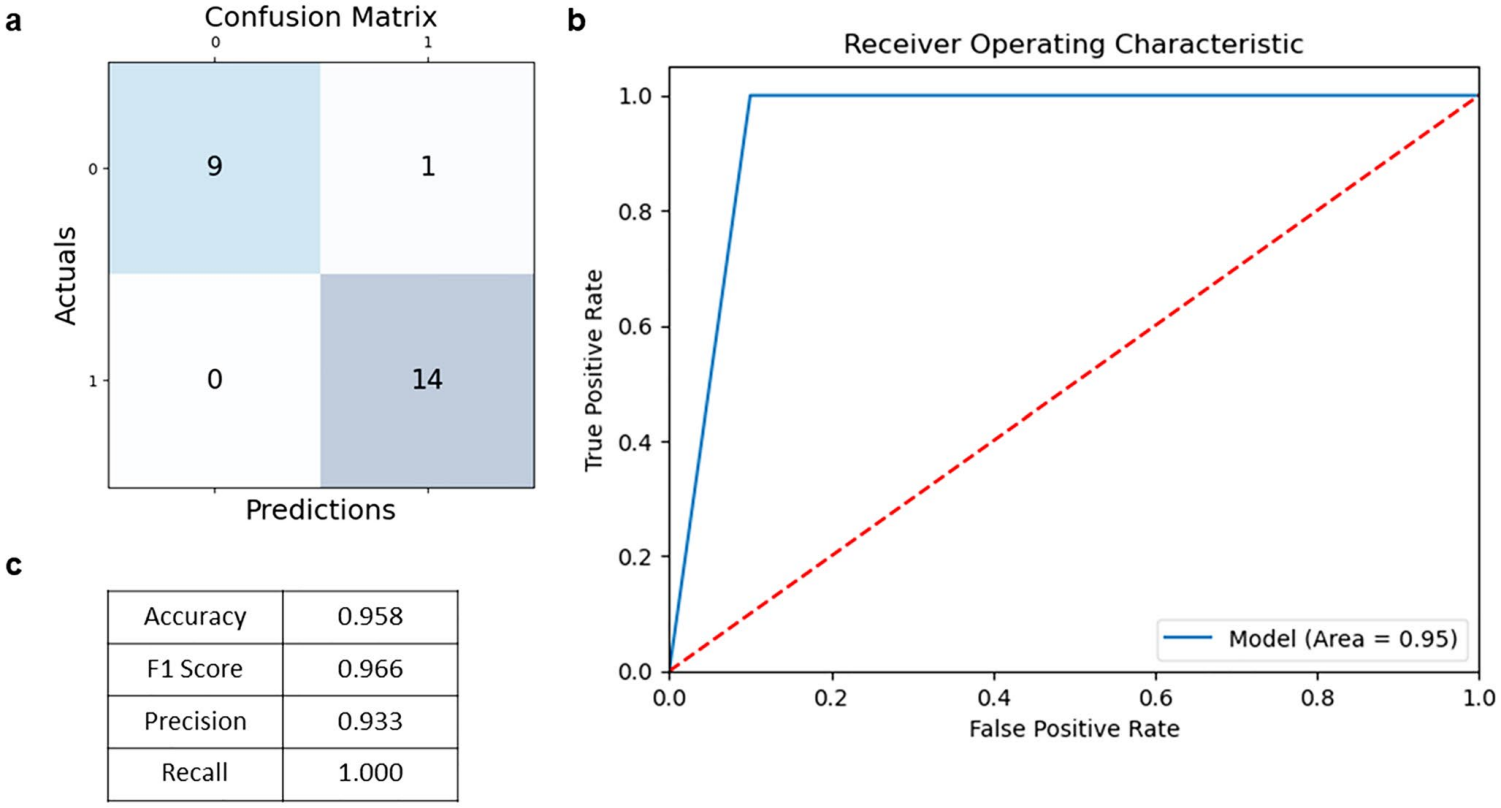
Evaluating the performance of a supervised machine learning algorithm. **a** Confusion matrix; **b** accuracy, F1 score, precision and recall; **c** receiver operating characteristic (ROC) curve

ML model performance can be evaluated in a number of ways to determine the accuracy and effectiveness of the model in making predictions (James et al. 2021). One of the most fundamental tools for evaluating classifiers is a *confusion matrix*. In our RA exemplar (Fig. 3a), the confusion matrix shows the number of true positive (TP), false positive (FP), true negative (TN) and false negative (FN) predictions made by the model in the test split. This information already indicates areas where model performance could be improved — if the model has a high number of false positives, it may be too aggressive in predicting the positive class, and a potential improvement could be to adjust the decision threshold. Alternatively, if the model has a high number of false negatives, it may be missing important features or require more data to improve its performance. The confusion matrix values are used to calculate *accuracy*, *F1 score*, precision and recall (Fig. 3b).

Accuracy is the number of correct predictions made by the model divided by the total number of predictions made. When classes are balanced and all errors are equally costly, this is the optimal measure of model performance. However, accuracy can be a misleading measure when these assumptions are violated. For instance, consider a setting in which 95% of samples are healthy and 5% are diseased. In this case, an invariant classifier — i.e. one that simply returns the prediction “healthy” no matter what — will be 95% accurate, which may sound impressive. In fact, the model is completely uninformative. Even when classes are balanced, we may want to assign different penalties to false positives and false negatives. For example, labelling a healthy patient as diseased may not be nearly as risky as labelling a diseased patient as healthy.

In light of these considerations, alternative measures are often used to evaluate classifiers. These include the precision (true positives as a proportion of predicted positives), recall (true positives as a proportion of actual positives) and F1-score (the harmonic mean between precision and recall). Overall performance can also be summarised using the *receiver operating characteristic (ROC) curve* (Fig. 3c), which shows the relationship between the true positive rate and the false positive rate of a model at various classification thresholds and can be used to evaluate the overall performance of a model (James et al. 2021). Alternatively, the precision–recall curve may be more appropriate for imbalanced data (Sofaer et al. 2019).

The performance of our XGB machine learning model in our RA exemplar is shown in Fig. 3. The performance metrics are very high. Although there is clear separation of case and control blood samples in PCA and t-SNE analysis, the performance is still likely to be misleading, given the small size of the dataset (70%/30% training/test splits of 31/14 RA cases and 24/10 controls), which is likely to lead to overfitting and other false discovery issues discussed below. This exemplifies the challenge of biomarker discovery. In this case, the study by Tasaki et al. in *Nature Communications*, reports on 45 RA cases and 34 controls. These sample sizes are typical for biomarker studies and probably reflect what is achievable within a “standard” research funding envelope over several years. Power calculations are important to ensure the adequacy of sample size, but these are often not performed in exploratory analyses, and indeed, exploratory analyses may be needed to obtain a realistic estimate of required power (ref). In this case to evaluate the model’s generalisation performance, a separate, sufficiently powered validation dataset that is representative of the same RA phenotype would be needed, which is also acknowledged by the authors of the original study (Tasaki et al. 2018).

## Hyperparameter tuning and comparative optimisation of ML models

Optimisation of a ML algorithm is important to identify the best set of configuration settings to maximise performance, a process known as hyperparameter tuning. Hyperparameters may be fixed before training or selected using a validation set (distinct from training and test sets). They can significantly affect model performance, making tuning an important step in the pipeline. Tuning can be done manually, which is time-consuming, or automatically using optimisation techniques such as grid search, random search or Bayesian optimisation, which may lead to better performance (refs). To keep our RA exemplar simple, we have not performed hyperparameter tuning, although this would always be recommended to ensure the optimal output of a model.

Beyond optimisation of individual algorithms, the choice of algorithm may also be key. LazyPredict is a Python package for comparing and evaluating machine learning models on a given dataset (https://github.com/shankarpandala/lazypredict). It allows for quick and efficient model selection without the need for extensive parameter tuning or feature engineering, supporting a wide range of algorithms for both classification and regression problems, and includes evaluation metrics for each. Users provide a labelled dataset and specify the problem type, and the package applies a range of models, outputting results sorted by performance. While packages like LazyPredict and other AutoML tools can be a great starting point, these methods do not replace the need for careful feature engineering and model selection and should only be used as a starting point for more in-depth analysis and experimentation. We have not included any algorithmic benchmarking in our RA exemplar, but this could potentially improve the model prediction further.

## Towards robust, validated biomarker discovery

ML methods have greater versatility and power than conventional statistical methods. However, they do not escape the fundamental challenges of biomarker discovery. When faced with increasing numbers of variables or dimensions, the likelihood of discovering spurious associations increases. In omics, where sample sizes are frequently limited but datasets often include millions of features, we are often in the high-dimensional regime, where feature *p* far exceed samples *N*, i.e. *p* >  > *N* (Hastie et al. 2001). High dimensionality presents obstacles for both statistical analysis and ML, but the problems it causes arise from different sources and have distinct consequences, even though the end result — failure to validate — may seem the same. Hypothesis testing and model building are very different processes which should not be conflated, as arguably they define the difference between classical statistics, where inference is made on parameters, and machine learning, where models are essentially prediction engines.

In a classical frequentist setting, multiple testing can lead to *false discovery*. This is because we typically set a target false positive rate (conventionally 5%) and declare all *p*-values below this threshold to be positive. Under the global null hypotheses that all *p*-values are uniformly distributed, we would therefore expect 5% of our tests to be false positives. With tens of thousands or perhaps even millions of tests, this produces an avalanche of false alarms. Simply lowering our threshold can help, but it is not immediately obvious what would constitute an appropriate cut-off in different settings. For instance, Bonferroni correction (Noble 2009) can be overly conservative in omics studies, successfully controlling the false positive rate but potentially inflating the false negative rate to unacceptable levels. An alternative error control target is the false discovery rate (FDR), i.e. the proportion of declared positives that are false (Benjamini and Hochberg 1995). FDR strikes a more reasonable balance between sensitivity and specificity and has become a popular indicator of significance in omics studies.

ML methods can also introduce a different source of failure in efforts to validate findings, particularly when trained on large datasets where they can identify complex patterns in the data, which may not be replicated in independent validation datasets. This is known as *overfitting*, where the model is too closely tuned to the training data and performs poorly on new data (Demšar and Zupan 2021). ML models can be particularly prone to overfitting when the number of features or predictors is very large compared to the sample size. This is the norm in most biomarker discovery studies as discussed above. Additionally, machine learning models can be sensitive to the presence of outliers or noise in the data, which can lead to false discoveries. This is particularly true for unsupervised methods such as clustering, where the presence of outliers can lead to the creation of additional clusters or groups.

Many methods have been proposed to control overfitting in machine learning. These include regularisation techniques (Acharjee 2012) and the use of cross-validation to evaluate the performance of the model (Bates et al. 2021). We highlight in particular one popular supervised learning approach called boosting, which uses “the wisdom of crowds” to train models sequentially (Freund and Schapire 1997). On each new iteration, sample weights are updated to give more importance to data points that the previous model misclassified, combining multiple weak learners into a single strong learner. Boosting has some desirable theoretical properties and has shown excellent performance in a wide variety of applications (Friedman 2001; Bühlmann and Yu 2003). However, boosting can also lead to overfitting if the number of iterations is too high and can be computationally expensive. Extreme gradient boosting (XGBoost) is a popular implementation with well-maintained Python and R libraries. XGBoost includes many features that make it well-suited for biomarker discovery studies, such as built-in regularisation and efficient cross-validation procedures for hyperparameter tuning. We apply the XGBoost model in our exemplar.

## Explaining and interpreting the output of machine learning models

Although supervised learning techniques have many benefits and have achieved impressive results, one major concern is their interpretability. Most successful ML models are so complex that it would be impossible for a human to understand how they make individual predictions. These models may have thousands or millions of parameters and involve a long series of recursive nonlinearities, making them effectively a “black box” that is difficult to understand or trust. This can be a barrier to using these models to gain insight into biological processes or ultimately applying them in the clinic (Watson et al. 2019).

*Explainable AI (XAI) or interpretable machine learning* is a rapidly growing research area focused on resolving the black box problem with human interpretable explanations (Molnar 2019) (https://christophm.github.io/interpretable-ml-book/). This gives an opportunity for biologists to scrutinise the findings of ML models and often the combination of human and machine intelligence can lead to further insight, improving prospects for biomarker discovery and importantly illuminating potential mechanisms that can be further explored in the lab and hopefully replicated in other populations. We argue that the potential for ML to provide explanation of mechanism is an often overlooked benefit of these methods beyond their primary role in prediction.

There are two different approaches to XAI. In the first instance, *intrinsic XAI methods* are built transparently into the model from the start and do not require additional explanation after the fact. Prominent examples include linear and logistic regression or decision trees. These algorithms have interpretable parameters that correspond to meaningful quantities, such as a log fold change for a given biomarker. However, when using complex alternatives such as deep neural networks, post hoc* XAI methods* are required to explain predictions. Different methods may produce different types of output, such as feature attributions, visualisations, decision rules or natural language explanations. Explanations may also be at a *global* level, providing an overall understanding of model behaviour, or at a *local* level, providing an understanding the predictions made for specific input data.

In most cases, particularly in complex genomic analysis, XAI is applied post hoc as the complex algorithms required to detect a biologically significant patterns are by nature very complex and thus defy human inspection (Watson 2022). But intrinsically explainable models could be appropriate where data inputs are simple and low dimensional, based on well-established concepts. This is perfectly illustrated by the PREDICT tool for breast cancer prognosis (Wishart et al. 2010), where 10 common clinical measures are employed to estimate the risk of breast cancer recurrence and death over a 10-year period using a Cox proportional hazards model (https://breast.predict.nhs.uk/tool). By contrast, a random forest model was used to predict breast cancer risk using the PAM50 panel based on expression of 50 genes (Parker et al. 2009). The researchers trained the model on gene expression data from breast tumours and normal breast tissue, along with clinical data such as age and tumour size. In this case, post hoc XAI would be needed to explain the output of the model, although the authors chose to accept the model performance on the basis of robust validation in independent samples. Others have applied post hoc explanation to predictions made on the PAM50 model. In one case (Zhang et al. 2021), researchers used the SHAP package to explain the output of a highly complex graph convolutional neural network. We explore the SHAP package in some detail below.

Our focus here on the explainability of machine learning and AI may seem esoteric, but there are many reasons why we may want to understand how machine learning algorithms make predictions. Some of these are important for downstream clinical applications, for example, to *audit for bias*, avoiding the perpetuation of social inequalities in healthcare such as underrepresented groups in medical research (Goh et al. 2017) or to ensure transparent decision-making on the basis of potentially sensitive attributes, such as gender (Gemmati et al. 2019). Beyond immediate issues of transparency and bias, explainability may be critical in omics analysis by merit of the *mechanistic insight* that it can potentially bring. We would argue that this is one of the most overlooked properties of XAI, potentially with dual benefits. On the one hand, it presents an opportunity for novel understanding of biology and disease processes. At the same time, mechanistic insight may also offer biologically plausible findings that are more likely to be independently validated. It might be argued that this latter property could ameliorate the issues of power and overfitting that hamper most omics biomarker discovery.

Of course, XAI tools cannot provide causal information on their own. This requires strong assumptions and/or background knowledge about the data generating process. Machine learning models themselves — specifically, empirical risk minimisation algorithms such as standard neural networks and gradient boosting machines — rely on observed associations alone. But these can be inconclusive with regard to causal mechanisms, since correlations may be spurious or confounded. Some critics argue that dressing ML models up with explainability methods can deceive patients and doctors into misunderstanding complex biological processes(Babic et al. 2021). However, with even minimal background knowledge (e.g. a partial ordering over variable classes), we can use XAI tools to quantify causal impact and disambiguate direct from indirect effects (Loftus et al. 2023). Moreover, these methods can integrate with existing procedures to generate new hypotheses that can be tested using more traditional methods such as gene knockout experiments.

## Building mechanistic insight with prior knowledge and XAI

As we have discussed, ML algorithms are prone to overfit solutions, especially with small sample sizes. Prior knowledge can help identify or remove spurious signals both as an input feature (Culos et al. 2020) or at the interpretation stage (Ideker et al. 2011). Alongside the context that prior knowledge can bring to an explanation, it can also add confidence to novel mechanisms, which exist in the context of known mechanisms. A good illustration of this approach is seen in Belyaeva et al. (2021), where an elastic net algorithm was used to construct causal network models of SARS-CoV-2 expression and aging, which were based on the integration of multi-omics data. These models were then integrated with prior knowledge of biological pathways to identify potential drug targets for COVID-19 treatment that were more likely to be validated based on existing experimental evidence and clinical trial results.

## Quantifying explainability with prediction metrics

ML models generate a range of metrics that can help to understand the basis on which a model is making a prediction. For instance, *variable importance* (VI) is a measure that indicates how much a specific feature contributes to a model’s prediction. Many algorithms come with internal VI measures, such as the coefficients of a linear model or the permutation importance of random forests. More generic post hoc methods are required for other function classes, such as neural networks. Calculating VI can be computationally intensive and may also pose challenges when applied to large datasets with many features.

*Local linear approximators* are another type of XAI tool that provide explanations for individual predictions. Local explanations can reveal which factors were most important in making the prediction by assigning scores to input features that quantify their relative contributions, which can be useful in understanding mechanism. Local linear approximators are widely used for computing local explanations and where VI measures may be less meaningful for models analysing non-linear interactions or non-additive combinations of features, such as tree-based models or neural networks. Local explanations may perform better by assigning weights to each input feature that sum to the model output. Widely used examples of local linear approximators include LIME (Ribeiro et al. 2016) and SHAP (Lundberg and Lee 2017), both of which are implemented in popular Python libraries.

**Fig. 4:**
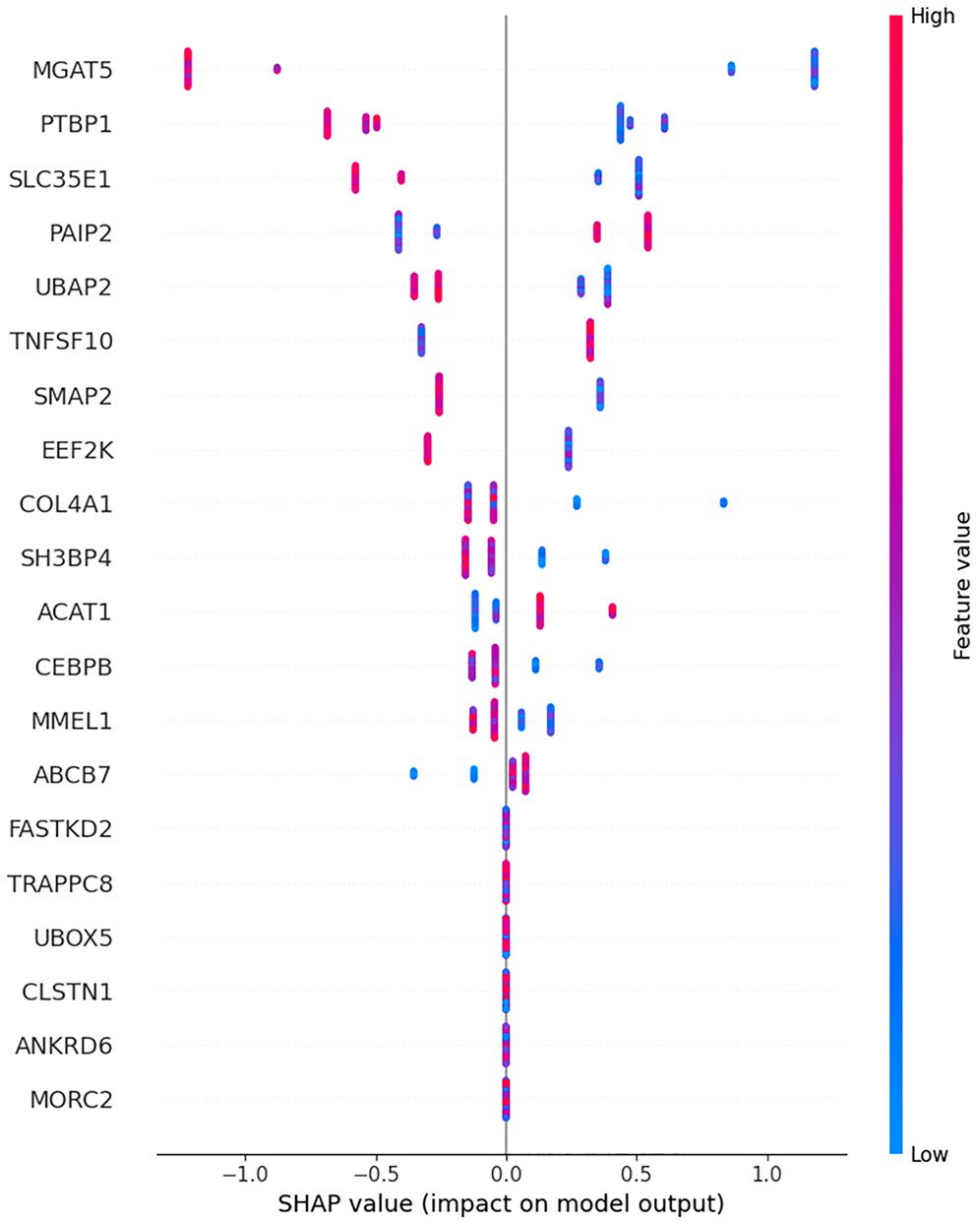
SHAP summary plot. Genes with prior support in RA in the open-target resource are indicated with *

As an example of these XAI tools, in our RA exemplar, we focus on the SHAP (SHapley Additive exPlanations) package, which explains model output using a local linear approximator that assigns a feature importance score to each input variable, in this case gene expression. A SHAP summary plot (Fig. 4) is a visual representation of these feature importance scores, which can help to interpret the model’s predictions and understand the relationships between the input variables (genes) and the output (prediction of case status). While the results from SHAP are not necessarily definitive, they offer a unique and principled approach for ranking and selecting features (in this case genes) and exploring interactions that can provide new insights and guide future experiments. The multivariate nature of these attributions makes them more informative than gene-level analyses commonly used in differential expression testing. Additionally, they may be more meaningful at an individual patient level because SHAP explains specific predictions rather than average behaviour throughout a feature space. Local linear approximators, which are flexible and based on standard game theoretic axioms, are a valuable tool for genomic research, but it is important to note that the importance of a feature in a model does not necessarily reflect its importance in nature.

Looking closely at the SHAP summary plot in Fig. 4, the horizontal bars display SHAP values for each input gene feature, sorted in descending order by absolute value. Positive SHAP values indicate that the corresponding feature increases the predicted output value, in this case prediction of RA case status, while negative SHAP values indicate that the feature decreases the predicted output value. The length of the bar represents the magnitude of the SHAP value, and the colour indicates the direction of transcript expression, blue represents lower expression in cases, and red represents higher expression in cases. For example, MGAT5 shows consistently lower expression in RA cases than controls, while PAIP2 and TNFSF10 are consistently higher expressed.

To interpret a SHAP plot, start by identifying the most important features, which are the ones with the largest absolute SHAP values. These features have the greatest impact on the model’s predictions and are likely to be the focus of further analysis. A quick way to quantitatively evaluate genes for a known phenotype association is to perform a query in the Open Targets database (Ochoa et al. 2021), which uses a weighted harmonic sum of association scores across a range of data sources. In this case, Open Targets ranks five genes in the SHAP plot with RA. These genes include with score 0–1 in parentheses MGAT5 (0.002), TNFSF10 (0.0458), ACAT1 (0.1008), CEBPB (0.0269) and MMEL1 (0.3471). Even though the data originates from blood rather than disease tissue, these results are consistent with RA biology. MGAT5 is a glycan-modulating enzyme, associated with several autoimmune diseases, which has been shown to fine-tune inflammation by regulation of cellular glycosylation, instructing both pro-inflammatory and anti-inflammatory responses (Alves et al. 2022). TNFSF10 showed differential expression in RA and decreased following anti-TNF treatment (Wang et al. 2022). ACAT1 has been consistently identified in genetic association studies of RA (Okada et al. 2014). CEBPB plays a role in chronic inflammation of the synovium in RA (Pope et al. 1999). Membrane metallo-endopeptidase-like (MMEL1 1) is most high ranked by Open Targets, also known as neprilysin 2, while MMEL1 is a membrane-bound metalloprotease that has been implicated in a range on autoimmune disease, including RA (Mathebula et al. 2022).

Even though the SHAP ranked genes are generally plausible candidates, notably, for those genes that are ranked, the Open Targets RA association rank and the SHAP rank are generally inverted. This highlights the need to critically appraise the output of a SHAP plot. One way to do this is to look for patterns in the SHAP values across different input values or samples. Are there certain features that consistently have positive or negative SHAP values across all or most samples? We can also consider how the SHAP values relate to the actual values of the input variables. Are there any unexpected or counterintuitive relationships between the SHAP values and the input values, for example, the direction and magnitude of expression changes? For the RA candidates, most effect directions are as expected. For example MGAT5 knockout causes an autoimmune phenotype in mice (Alves et al. 2022), which is consistent with the downregulation observed in RA patients. Insights based on prior knowledge can help to validate the model’s performance or provide new insights into the underlying data. The consistency of a feature can provide important information on the mechanistic nature and performance of the model. Most genes show at least some inconsistent expression trends in one direction; some are widely inconsistent (e.g. PAIP2 and COL4A1). Consistent features are likely to be more reliable predictors and can be used to identify important patterns or relationships in the data, while inconsistent features may require further analysis or may indicate a weakness in the model, perhaps due to overfitting.

**Fig. 5:**
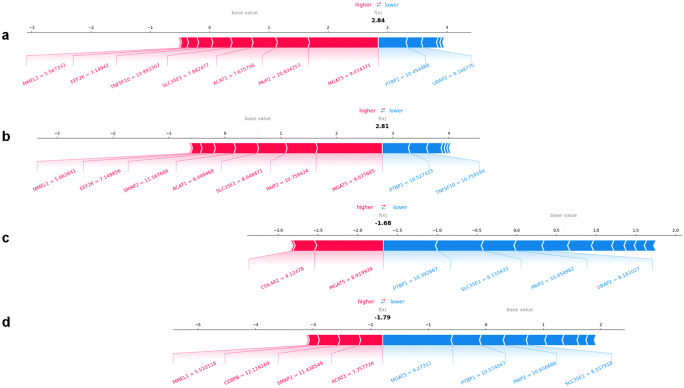
FORCE plots for two RA patients (**a**, **b**) and two healthy controls (**c**, **d**)

While SHAP plots do show individual patient data points for each feature, they do not show clearly how features interact at an individual level. A force plot is a visualisation tool that is commonly used in conjunction with SHAP values to explain the output of a model. In Fig. 5, the force plot displays the individual contributions of each gene feature to the prediction in four individuals. Two RA patients in 5A and B are correctly predicted cases and 5C and 5D are correctly predicted healthy controls.

A typical force plot consists of a series of nodes that represent the input features, connected by arrows that indicate the direction and magnitude of the feature’s impact on the output. The length and colour of the arrows represent the SHAP values for each feature, with longer arrows indicating larger SHAP values. At the top of the force plot is the base value, which represents the average or baseline prediction of the model. The sum of the SHAP values and the base value equals the actual prediction of the model for the given input. To interpret a force plot, start by identifying the most important features, which are the ones with the longest arrows. These features have the greatest impact on the model’s prediction and should be the focus of further analysis. Next, consider the direction of the arrows, which indicates whether the feature is increasing or decreasing the predicted output value. Features with arrows pointing to the right are increasing the output, while features with arrows pointing to the left are decreasing the output. Finally, look for interactions between features, which are indicated by the way the arrows are connected. If two features have arrows pointing in opposite directions but are connected by a common feature, it may indicate an interaction or trade-off between the two features.

A force plot can help to understand how the input features interact to influence the prediction of the model. A tug of war may be a useful analogy for a force plot, particularly in inflammatory diseases like RA, where pro-inflammatory genes in red on the left-hand side of the plot interact against anti-inflammatory genes in blue on the right side of the plot. Intriguingly, though, some gene roles are more consistent than others. For example, in 5A, TNFSF10 is upregulated, but in 5B, it is downregulated, showing that regulation of the gene may be context dependent. Similar trends are seen for most genes. This may highlight how simple definition of up- and downregulated genes or panels of genes may have limited utility as biomarkers.

Conceptually, it could be argued that force plots are a promising way to present patient endotypes, as they are able to capture the nuanced balance between a set of genes in the context of an individual patient. It might be possible to use a force plot as the basis of a disease endotype that could be used to define an individualised treatment strategy. This is certainly worth further research, but there are several limitations to force plots in this context that must also be considered. First, force plots only show the contributions of individual features to the model’s output and do not capture interactions or correlations between them. This means that important features that are dependent on other features may not be highlighted in the force plot, and the relationships between features may be oversimplified (Lundberg et al. 2020). Force plots also rely on the accuracy and interpretability of the underlying ML model. If the model is overfit or poorly calibrated, the force plot may not accurately reflect the true contributions of the features to the disease phenotype. This emphasises that they should be used with caution and in conjunction with other analysis techniques to ensure that the results are accurate and meaningful in the context of the available data and prior knowledge of the disease.

## The future of XAI and genomics

Given the recent hype surrounding the rise of large language models (LLMs) like ChatGPT (van Dis et al. 2023), it is tempting to speculate on the possible role of this relatively new form of AI in explainability for biomarker discovery and research more generally. One of the most potentially powerful properties of LLMs is their ability to mine a large corpus of text data for potentially relevant associations. Sometimes, the model can generate responses that are not supported by the input data or the prior information the model has been trained on. This is known as *hallucination* and can occur when the model overfits to the training data or when the input is ambiguous or incomplete. Hallucination can be a big challenge for LLMs, as it leads to inaccurate or misleading responses that do not reflect the true meaning or intent of the input. In the case of ChatGPT, given a query like “what mechanism links MGAT5 to rheumatoid arthritis pathology,” the following response is provided with several supporting references: “Studies have shown that MGAT5 is upregulated in the synovial tissue of patients with RA, and that this upregulation is associated with increased inflammation and joint damage. It has been suggested that this may be due to alterations in the structure of N-glycans on the surface of immune cells in the synovial tissue. Specifically, the increased expression of MGAT5 in RA may lead to the production of N-glycans with more branched structures, which in turn may enhance the binding of immune cells to inflammatory molecules, such as cytokines and chemokines, leading to increased inflammation and joint damage.” On closer examination, the references do not exist, and the specific statements made are generally not supported specifically in RA, but rather the statements generalise across a range of IMIDS including SLE and MS.

While hallucination in LLMs like ChatGPT is generally acknowledged to be a problem, there may be potential benefits or use cases where it could be advantageous or desirable. In the case above, the LLM is taking what it knows about MGAT5 in other diseases and generalising it to RA. This is effectively a creative writing exercise, which goes beyond the constraints of the input or prior information to explore a hypothetical scenario. In the MGAT5 case, the scenario is highly plausible, and it could be argued is worthy of further investigation.

But currently, ChatGPT’s scientific utility remains limited, with much less access than Google Scholar, for example. The curse of dimensionality may apply equally to LLMs. Research-focused implementations of similar language models may be more appropriate and are also starting to appear (Luo et al. 2022). However, even ChatGPT has access to gene databases such as GenBank, Ensembl and Gene Ontology which provide information on gene sequences, structures and functions. It also has access to the KEGG and Reactome pathway databases, as well as the GEO and ArrayExpress gene expression databases. On a practical level, this means that LLMs could effortlessly connect genes with multiple degrees of separation, drawing inferences based on pathway, expression and functional information. On this basis, it seems likely that this capability will soon be applied to the output of ML models, both to provide explanations and potentially to rank model output on the basis of the most plausible explanation. The impact of such capabilities could be profound, with an opportunity to exponentially increase knowledge. However, these potential gains are not without risk, as existing biases could become further entrenched. Perhaps of greater concern, we could see a regression towards the mean in knowledge creation, which could suppress opportunities for paradigm shifts in innovation.

## Conclusion

As the field of genomics begins to mature, AI has increasingly come to the fore as an essential partner for genomic data interpretation. Significant progress has been made in AI, and XAI has already revealed novel insights in many genomic studies. XAI can serve researchers in various ways, including auditing models for bias, validating performance before and during deployment and revealing novel mechanisms for further exploration. A number of challenges remain. However, as research in supervised learning and genomics continues to evolve, XAI will continue to become an integral part of the genomics toolkit and standard research practice, helping to generate and test hypotheses as well as train and deploy models. The advent of high-throughput sequencing has made ML methods indispensable for modern bioinformatics. Now the challenge moves to the clinical investigators to scale cohort recruitment to enable replication of findings and to funders to make these studies possible.

## Footnotes

1 A third major branch of ML is reinforcement learning, where an agent must navigate an environment to optimise rewards. We restrict our focus in this article to supervised and unsupervised approaches, which are more common in bioinformatics.
